# Deep Learning-Based Classification of Canine Cataracts from Ocular B-Mode Ultrasound Images

**DOI:** 10.3390/ani15091327

**Published:** 2025-05-04

**Authors:** Sanghyeon Park, Seokmin Go, Seonhyo Kim, Jaeho Shim

**Affiliations:** 1 Helix Animal Medical Center, Seoul 06546, Republic of Korea; sanghyun0218@snu.ac.kr; 2 Nowon N Animal Medical Center, Seoul 01704, Republic of Korea; resume314@hanmail.net; 3 Ilsan Animal Medical Center, Ilsan 10368, Republic of Korea; vetophyo@gmail.com; 4 Institute of Animal Medicine, College of Veterinary Medicine, Gyeongsang National University, Jinju 52828, Republic of Korea; 5 Department of Veterinary Ophthalmology, College of Veterinary Medicine, Gyeongsang National University, Jinju 52828, Republic of Korea

**Keywords:** canine cataract, ultrasound imaging, veterinary ophthalmology, deep learning, convolutional neural network, artificial intelligence, diagnostic imaging

## Abstract

Cataracts are a prevalent cause of vision loss in dogs, and timely diagnosis is essential for effective treatment. This study aimed to develop and evaluate deep learning models to automatically classify canine cataracts from ocular ultrasound images. A dataset of 3155 ultrasound images (comprising 1329 No cataract, 614 Cortical, 1033 Mature, and 179 Hypermature cases) was used to train and validate four widely used deep learning models (AlexNet, EfficientNetB3, ResNet50, and DenseNet161). Data augmentation and normalization techniques were applied to address category imbalance. DenseNet161 demonstrated the best performance, achieving a test accuracy of 92.03% and an F1-score of 0.8744. The confusion matrix revealed that the model attained the highest accuracy for the No cataract category (99.0%), followed by Cortical (90.3%) and Mature (86.5%) cataracts, while Hypermature cataracts were classified with lower accuracy (78.6%). Receiver Operating Characteristic (ROC) curve analysis confirmed strong discriminative ability, with an area under the curve (AUC) of 0.99. Visual interpretation using Gradient-weighted Class Activation Mapping indicated that the model effectively focused on clinically relevant regions. This deep learning-based classification framework shows significant potential for assisting veterinarians in diagnosing cataracts, thereby improving clinical decision-making in veterinary ophthalmology.

## Simple Summary

Cataracts are a common eye disease in dogs that causes clouding of the lens and loss of vision. Early diagnosis helps veterinarians provide better treatment; however, distinguishing different stages of cataract using ultrasound images could be subjective in some cases. This study developed a computer-assisted method using artificial intelligence to automatically classify canine cataracts from ultrasound images. Among several tested computer models, one showed excellent accuracy in distinguishing cataract stages. This approach could help veterinarians to quickly and accurately identify cataracts, improving the quality of eye care for dogs.

## 1. Introduction

Cataracts are among the most prevalent ocular disorders in canine patients, characterized by progressive lens opacification that frequently leads to vision impairment and potential blindness, and they constitute a significant proportion of intraocular pathologies encountered in veterinary ophthalmology practice [1,2,3]. Accurate diagnosis and timely intervention are essential to preserve visual function and quality of life [1,2]. However, the complexity of cataract staging and management necessitates evaluation by experienced clinicians to achieve optimal patient outcomes [1,4].

B-mode ocular ultrasonography is a widely used, cost-effective, and non-invasive imaging technique that provides detailed cross-sectional images of the eye [1,5,6]. By detecting echoes reflected from various ocular tissues, this modality enables clinicians to evaluate intraocular and retrobulbar structures [2,5]. Consequently, ultrasonography has become an essential diagnostic tool for various ophthalmic conditions, including cataracts, intraocular tumors, and retinal detachment, thereby facilitating diverse therapeutic strategies [1,6,7]. Due to these advantages, ultrasonography remains indispensable in general veterinary practices where access to advanced ophthalmic imaging modalities and specialized ophthalmologists may be limited [2,7]. Despite its utility, B-mode ultrasound images are limited by acoustic artifacts and relatively low resolution, which can hinder accurate interpretation. Interpretation also remains subjective, as diagnostic accuracy depends on the examiner’s experience and skills, unlike modalities offering objective, quantitative data [6].

Historically, cataract diagnosis using computer vision relied on manual feature engineering, which was labor-intensive and lacked scalability [1]. Recently, convolutional neural networks (CNNs) have overcome these limitations by learning hierarchical feature representations directly from raw images, thereby enhancing classification accuracy and generalizability [8]. CNNs, a subset of deep learning, have demonstrated remarkable potential in medical image analysis by automatically extracting critical features from images [8,9,10]. Their architecture, composed of convolutional and pooling layers, enables efficient feature extraction and pattern recognition [11,12]. However, despite these advantages, CNNs are often criticized as “black-box” models due to their limited interpretability, posing challenges for clinical adoption where transparency is crucial [8].

To address the interpretability challenge, techniques such as Gradient-weighted Class Activation Mapping (Grad-CAM) have been developed [7,10,13]. Grad-CAM provides visual explanations for model predictions by highlighting key areas in an image that influence decision-making [13,14]. This technique offers partial insight into the reasoning behind model outputs and has been widely adopted in CNN-based studies to enhance interpretability and promote clinical acceptance [13].

Although there have been substantial advancements in AI applications for imaging in veterinary clinics [9,10,15,16], research specifically focusing on AI-assisted analysis of ocular ultrasonography remains limited. Further development in this area could significantly enhance diagnostic accuracy and clinical decision-making in veterinary ophthalmology. Therefore, the aim of this study was to determine whether CNN models could accurately classify canine cataracts into four maturation stages (No cataract, Cortical cataract, Mature cataract, and Hypermature cataract) using B-mode ultrasound images, and to comprehensively evaluate their diagnostic performance and clinical applicability.

## 2. Materials and Methods

### 2.1. Datasets

We employed ocular B-scan ultrasound images sourced from the publicly accessible AI-HUB platform (www.aihub.or.kr, accessed on 2 January 2025). The dataset is publicly available upon request via AI-HUB, subject to compliance with their usage terms and conditions. A total of 3155 ultrasound images were collected and categorized into four classes: No cataract, Cortical cataract, Mature cataract, and Hypermature cataract (Table 1). The mean age of all dogs was 8.9 ± 3.1 years. The cohort included 1644 females (52%) and 1511 males (48%). The most common breeds were Maltese, Poodle, Shih Tzu, and Yorkshire Terrier.

To enhance data quality and minimize redundancy, duplicate and visually similar images were identified and eliminated using VisiPics V1.3 software (https://visipics.info). In addition, images where the lens or globe structures were not clearly captured were excluded. Subsequently, all remaining images were manually reviewed and labeled by a veterinary ophthalmologist with a PhD degree to ensure accurate classification. Intra-observer and inter-observer reproducibility were evaluated by calculating agreement rates between repeated and independent classifications. Inter-observer reproducibility was assessed between the corresponding author and the first author (Sanghyeon Park, DVM, MS, veterinary ophthalmologist). The intra-observer and inter-observer agreement rates were 94.1% and 87.3%, respectively.

The dataset was stratified and randomly divided into training (80%), validation (10%), and test (10%) subsets to ensure that class distribution was preserved across all sets.

To improve the robustness of the evaluation, external ocular ultrasound images obtained from various veterinary clinics, including Gyeongsang National University Animal Medical Center, Helix Animal Medical Center, Ilsan Animal Medical Center, and Nowon N Animal Medical Center, were partially incorporated into the test subset (Table 1). The model’s performance was also separately assessed using only the external dataset. Images from the AI-HUB platform were designated as the internal dataset, while those collected from external veterinary clinics were designated as the external test dataset.

The dataset used in this study exhibited inherent class imbalance due to patient demographic characteristics, with certain cataract stages being more prevalent than others. Specifically, the No cataract category contained the largest number of samples (*n* = 1329), followed by Mature (*n* = 1033), Cortical (*n* = 614), and Hypermature (*n* = 179). Such imbalances may cause overfitting, leading the model to preferentially learn patterns from dominant classes and underperform on minority classes.

### 2.2. Data Augmentation and Model Development

To address class imbalance, data augmentation techniques were employed to enhance the representation of underrepresented classes and improve model generalization. Training images were modified through random rotations, horizontal flips, resizing, color jittering, Gaussian blurring, and random erasing to artificially expand the dataset and provide more diverse examples for training [17,18,19]. Additionally, class weights were computed using a balanced approach, assigning weights inversely proportional to class frequencies to ensure that minority classes contributed proportionally during model optimization [20].

To classify canine cataracts, four state-of-the-art deep learning models were utilized: AlexNet [21], EfficientNet-B3 [22], ResNet-50 [23], and DenseNet-161 [24]. AlexNet, one of the earliest CNN architectures, employs a sequential arrangement of convolutional and fully connected layers [1,21]. EfficientNet-B3 introduces a compound scaling method that uniformly adjusts network depth, width, and input resolution to optimize both accuracy and computational efficiency [22]. ResNet-50 incorporates residual connections that facilitate the training of deep networks by addressing the vanishing gradient problem [23]. DenseNet-161 employs densely connected layers to enhance feature reuse and gradient flow, improving learning efficiency and reducing the risk of overfitting [1,24].

A transfer learning approach was applied by initializing the models with pre-trained weights from the ImageNet dataset [25,26,27]. All layers of the networks were fine-tuned without freezing, allowing the models to fully adapt their feature representations to the specific characteristics of canine cataract ultrasound images [25,26,27].

The training process was conducted using a batch size of 64 and an initial learning rate of 1 × 10^−5^. Optimization was performed using the Adam optimizer [28] with a weight decay of 1 × 10^−4^, and a cosine annealing learning rate scheduler [29] dynamically adjusted the learning rate over 100 epochs. Early stopping [20] with a patience of 10 epochs was employed to prevent overfitting by halting training when validation performance plateaued. Hyperparameters were determined empirically by evaluating multiple combinations and selecting the configuration that provided the most stable validation performance.

**Figure 1:**
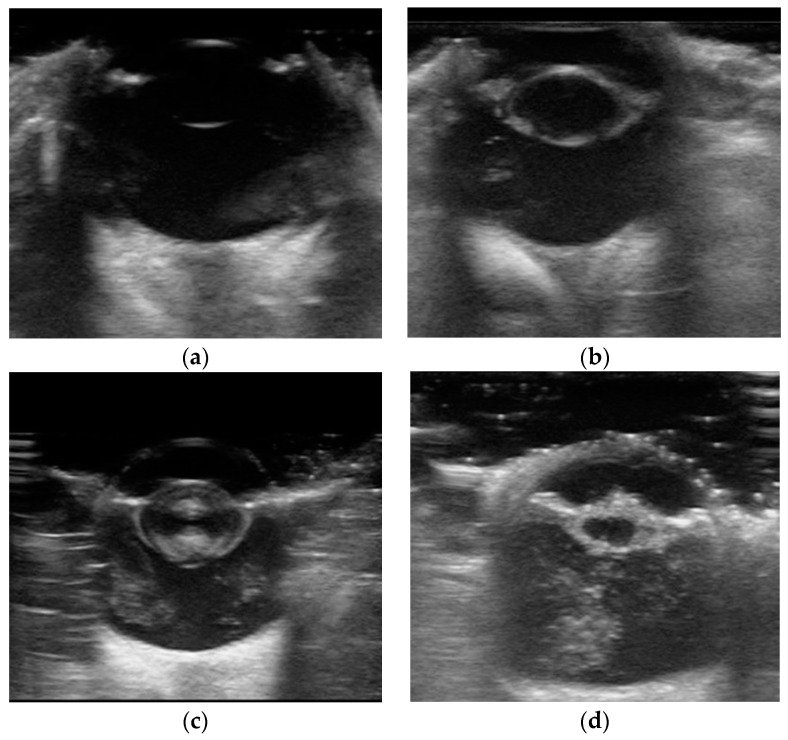
Representative samples from the collected eye B-ultrasound image dataset, showing (**a**) an eye without cataract, (**b**) an eye with cortical cataract, (**c**) an eye with mature cataract, and (**d**) an eye with hypermature cataract.

The lens status was categorized into four groups according to a previously described classification system [5]: No cataract, Cortical cataract, Mature cataract, and Hypermature cataract. Cortical cataracts were characterized by echogenic anterior and posterior cortices with clear visualization of the capsule. Mature cataracts exhibited enhanced echogenicity with asymmetry and near-complete lens opacification. Hypermature cataracts were identified by reduced axial thickness and wrinkling of the lens capsule (Figure 1).

### 2.3. Computational Environment

The model was implemented using PyTorch 2.5.1 with CUDA 11.8. Model training and evaluation were performed on a workstation equipped with an Intel Core i7-13700 CPU (Intel Corporation, Santa Clara, CA, USA) and an NVIDIA GeForce RTX 3060 GPU (NVIDIA Corporation, Santa Clara, CA, USA).

### 2.4. Evaluation Metrics

The classification performance of each CNN model was comprehensively evaluated using multiple metrics, including accuracy, F1-score, sensitivity, and specificity. To quantitatively compare diagnostic performance across different cataract stages, ROC curves were generated, and AUC values were calculated. Furthermore, Grad-CAM visualization was employed to highlight the discriminative anatomical regions utilized by each model during classification, thereby enhancing interpretability.

## 3. Results

### 3.1. Classification Performance on the Combined Internal and External Test Dataset

The classification performance of four CNN models was evaluated on cataract ultrasound images. Among the models, DenseNet-161 demonstrated the highest performance, achieving a test accuracy of 92.03% and an F1 score of 0.8744. In comparison, ResNet-50 achieved a test accuracy of 91.82% and an F1 score of 0.8553, EfficientNet-B3 achieved 89.52% and 0.8264, and AlexNet achieved 87.00% and 0.8086, respectively (Table 2).

### 3.2. External Validation Performance

The external validation performance of four CNN models was evaluated using an independent external dataset. Among the models, DenseNet-161 demonstrated the highest performance, achieving an external validation accuracy of 92.15% and a weighted F1 score of 0.9231. In comparison, ResNet-50 achieved an accuracy of 91.74% and a weighted F1 score of 0.9181, EfficientNet-B3 achieved 90.08% and 0.9064, and AlexNet achieved 84.71% and 0.8532, respectively (Table 3).

### 3.3. Confusion Matrix Analysis

**Figure 2:**
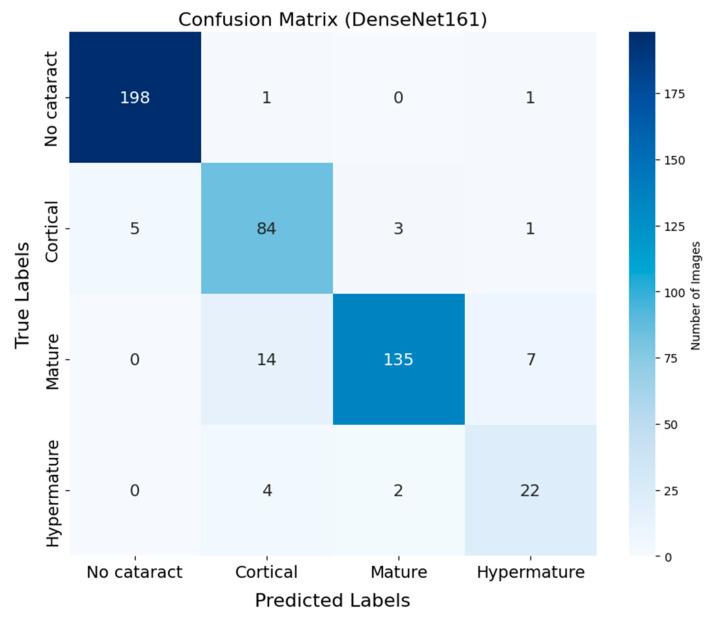
Confusion matrix of DenseNet-161 for cataract classification using ocular ultrasound images. The matrix illustrates the distribution of predictions across four categories: No cataract, Cortical, Mature, and Hypermature. Correct classifications appear along the diagonal, while off-diagonal values represent misclassifications. The No cataract category shows the highest accuracy, while some confusion is observed between the Cortical and Mature categories.

The confusion matrix of DenseNet-161 demonstrates the classification performance across four cataract stages (Figure 2). Correct classifications appear along the diagonal, while off-diagonal values represent misclassifications. The No cataract category exhibited the highest classification accuracy, with 198 correctly classified instances out of 200 (99.0%), highlighting the model’s robustness in identifying eyes without cataracts. The Cortical category followed with 84 correct classifications out of 93 instances (90.3%), while the Mature category achieved 135 correct predictions out of 156 samples (86.5%). The Hypermature category showed relatively lower accuracy, with 22 correct predictions out of 28 samples (78.6%). Additionally, the sensitivity and specificity for each cataract stage were as follows: for No cataract, the sensitivity was 99.0% and the specificity was 99.4%; for Cortical cataracts, the sensitivity was 90.3% and the specificity was 97.4%; for Mature cataracts, the sensitivity was 86.5% and the specificity was 95.6%; and for Hypermature cataracts, the sensitivity was 78.6% and the specificity was 98.1%. The most common source of classification errors occurred between the Cortical and Mature stages, with 14 instances of Mature cataracts misclassified as Cortical and three instances of Cortical cataracts misclassified as Mature.

### 3.4. ROC Curve and AUC Analysis

**Figure 3:**
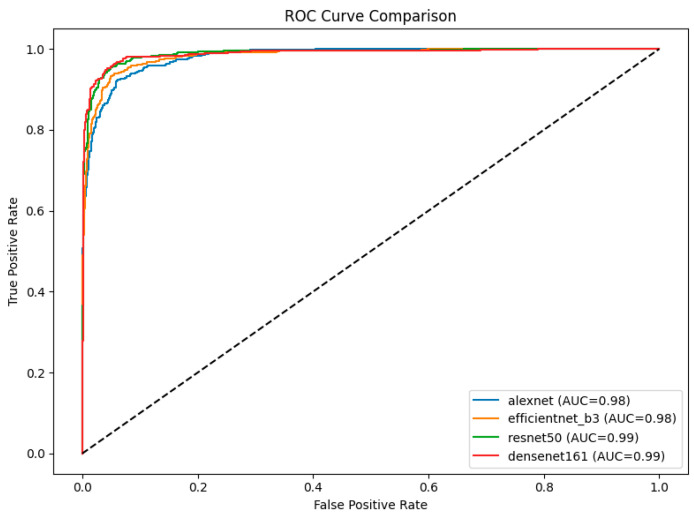
ROC curve comparison of four CNN models (AlexNet, EfficientNet-B3, ResNet-50, and DenseNet-161) on ultrasound images. The area under the curve (AUC) values indicated that ResNet-50 and DenseNet-161 achieved the highest AUCs (0.99), followed by EfficientNet-B3 and AlexNet (0.98).

The ROC curves for the four models provide a comparative analysis of their classification performance across cataract stages, with the AUC serving as a quantitative metric of their discriminative ability (Figure 3). Among the models, DenseNet-161 and ResNet-50 both achieved the highest AUC of 0.99, indicating superior classification performance and robustness in distinguishing between stages. EfficientNet-B3 and AlexNet followed with AUC values of 0.98 each. Notably, the ROC curves of DenseNet-161 and ResNet-50 consistently lie above those of the other models, underscoring their effectiveness in this classification task.

### 3.5. Model Interpretation Using Grad-CAM

**Figure 4:**
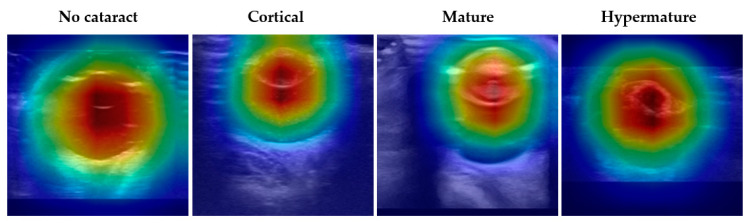
Grad-CAM visualizations illustrate the attention regions of DenseNet-161 for different cataract classifications in ocular ultrasound images. The columns represent the four categories: No cataract, Cortical cataract, Mature cataract, and Hypermature cataract. The red regions indicate areas of highest activation, demonstrating where the DenseNet-161 model focuses when making classifications. Across all categories, the model consistently prioritizes the lens region (red regions) while suppressing irrelevant background areas (blue regions).

To evaluate the interpretability of DenseNet-161, the best-performing model in diagnosing cataracts using ocular ultrasound images, Grad-CAM was employed to visualize the model’s focus during inference. The final convolutional layer was *used* for feature map generation. These visualizations reveal that the model predominantly attended to the lens region, as indicated by the red areas with high attention weights, while areas corresponding to irrelevant background appeared blue (Figure 4). This consistent focus on the lens across all categories underscores the model’s capacity to prioritize key anatomical features critical for accurate cataract diagnosis.

## 4. Discussion

In this study, DenseNet-161 demonstrated the highest classification performance among the tested architectures, achieving a test accuracy of 92.03% and an F1 score of 0.8744. The external validation set showed comparable model performance, with DenseNet-161 achieving an accuracy of 92.15% and a weighted F1 score of 0.9231. ROC curve analysis further confirmed the excellent discriminative ability of the model, with DenseNet-161 achieving an AUC of 0.99 across all cataract stages. These results indicate strong generalizability of the proposed approach across diverse clinical settings, despite potential differences in imaging protocols between institutions.

Although direct comparison is limited due to differences in evaluation metrics, as in a previous study, interobserver reproducibility among experienced investigators skilled in ocular ultrasonography ranged from r = 0.83 to 0.97 when interpreting B-mode ultrasound images in dogs [30]. This inherent variability highlights that interpretation inconsistencies exist even among skilled operators. Given this context, the 92.03% accuracy achieved by our model represents a promising level of consistency that could support objective cataract staging.

The confusion matrix revealed that most misclassifications occurred between the Cortical and Mature cataract categories. This pattern is likely due to their similar echogenic patterns, as cortical cataracts gradually progress toward the mature stage [5], introducing diagnostic ambiguity even among experienced observers [30]. These misclassifications might reflect the inherent subjectivity of intermediate-stage classification and are unlikely to substantially alter clinical management decisions.

Grad-CAM visualization demonstrated that the model consistently focused on the lens region, which corresponds with key anatomical landmarks assessed by ophthalmologists during cataract diagnosis using B-scan ultrasonography. By highlighting clinically meaningful areas, Grad-CAM improves the interpretability of the CNN model and enhances its diagnostic utility in veterinary ophthalmology.

In this study, we selected four CNN models based on their demonstrated effectiveness in medical imaging tasks and their structural suitability for analyzing grayscale B-mode ocular ultrasound images [8,31]. Although Vision Transformers (ViTs) have recently gained attention for various image analysis tasks, CNN-based architectures were prioritized due to their proven efficiency, robustness in limited-data settings, and practical suitability for structured image classification without requiring large-scale datasets [32].

Previous research reported a higher accuracy (98.01%) using a YOLO-v3 and DenseNet-161 combination for cataract detection [1]; however, YOLO-based methods require labor-intensive manual labeling of bounding boxes [33,34]. In contrast, our approach requires only single-label annotations for entire images, reducing workload while achieving a higher DenseNet-161 classification accuracy (92.03%) compared to prior studies (84.12%) [1].

High-quality imaging modalities, such as slit lamp systems, offer superior diagnostic capabilities but are often associated with high costs. In contrast, B-scan ocular ultrasonography is more accessible and widely available [1,35]. However, traditional ultrasound devices are limited by inherent subjectivity and low reproducibility, often resulting in diagnostic inconsistencies [6]. In this study, our CNN-based approach effectively mitigated these limitations, making early detection and treatment more feasible in resource-limited settings. Moreover, the integration of computer-assisted diagnosis helps reduce clinician workload and improve diagnostic efficiency, offering practical support, particularly in large-scale screenings and settings involving less experienced veterinary practitioners [6,7,36].

Transfer learning was utilized to improve model performance, particularly given the limited availability of veterinary ultrasound images compared to human datasets [34]. By leveraging pre-trained models, transfer learning enhances accuracy and generalization while reducing data and computational requirements [26,27]. This approach is particularly valuable in veterinary informatics, where data scarcity and lack of standardization present significant challenges [37].

Although the ultrasound acquisition protocols and transducer specifications were not standardized across all images, reliable cataract staging was achieved when critical anatomical structures, including the lens and globe, were clearly visualized. As demonstrated in our study, high classification accuracy could still be obtained despite minor variations in imaging conditions, supporting its applicability across diverse clinical settings.

Despite the strong classification performance demonstrated by the deep learning model, it cannot replace the final clinical diagnosis, which requires comprehensive integration of additional clinical information by a veterinarian [7]. Another limitation is the class imbalance within the dataset, primarily due to the limited number of Hypermature cataract cases, which may have biased the model’s performance [10,38,39]. Future research could improve class balance by collecting additional samples or by generating synthetic data using generative adversarial networks (GANs) [40]. Additionally, research could be expanded to classify a broader range of ocular conditions, including retinal detachment, vitreous degeneration, glaucoma, and various retinal diseases [13,41,42].

## 5. Conclusions

This study introduced a CNN-based model to classify canine cataracts using B-mode ocular ultrasound images, demonstrating substantial accuracy across four distinct categories: No cataract, Cortical, Mature, and Hypermature. The application of deep learning for cataract detection in veterinary medicine appears feasible, serving as a robust tool to enhance clinical decision-making. Additionally, these findings provide a foundation for future research aimed at diagnosing other ocular conditions, including retinal detachment, vitreous degeneration, glaucoma, and various retinal diseases.

## Figures and Tables

**Table 1:** Distribution of B-ultrasound images across training, validation, and test sets for each class.

Class	Training Count	Validation Count	Test Count	Total Count
No cataract	930	199	200	1329
Cortical	429	92	93	614
Mature	723	154	156	1033
Hypermature	125	26	28	179

**Table 2:** Classification accuracy and F1 score of four CNN models on the combined test dataset.

Model	Test Accuracy (%)	F1 Score
AlexNet	87.00	0.8086
EfficientNet-B3	89.52	0.8264
ResNet-50	91.82	0.8553
DenseNet-161	92.03	0.8744

**Table 3:** External validation performance of four CNN models.

Model	Test Accuracy (%)	F1 Score
AlexNet	84.71	0.8532
EfficientNet-B3	90.08	0.9064
ResNet-50	91.74	0.9181
DenseNet-161	92.15	0.9231

## Data Availability

The dataset used in this study was obtained from the AI-HUB platform (https://www.aihub.or.kr, accessed on 2 January 2025), which is supported by the Korean government. The data are publicly available for non-commercial research purposes through the AI-HUB portal.

## Abbreviations

The following abbreviations are used in this manuscript:

AI	Artificial Intelligence
ROC	Receiver Operating Characteristic
AUC	Area Under the Curve
Grad-CAM	Gradient-weighted Class Activation Mapping
CNN	Convolutional Neural Network
CPU	Central Processing Unit
GPU	Graphics Processing Unit
ViTs	Vision Transformers
YOLO	You Only Look Once
GANs	Generative Adversarial Networks
